# Willingness to pay for combined medical and old-age care services: A survey in Chengdu City, China

**DOI:** 10.1097/MD.0000000000045206

**Published:** 2025-10-17

**Authors:** Chao-Rong Guo, Yong-Guang Lu, Hui Zhu, Ye Zhao

**Affiliations:** a Department of Therapeutic Nursing, West China Second University Hospital, Sichuan University, Chengdu, Sichuan, China; b International College, Krirk University, Bangkok, Thailand; c Rehabilitation Department, West China Second University Hospital, Sichuan University, Key Laboratory of Birth Defects and Related Diseases of Women and Children (Sichuan University), Ministry of Education, Chengdu, Sichuan, China; d Out-patient Department, West China Second University Hospital, Sichuan University, Key Laboratory of Birth Defects and Related Diseases of Women and Children (Sichuan University), Ministry of Education, Chengdu, Sichuan, China; e Institute of Chinese Medicine, Yanting County People’s Hospital, Yanting, China.

**Keywords:** Chengdu, Jinjiang District, Willingness to pay

## Abstract

To provide a basis for service supply for older persons, we investigated older adults’ willingness to pay for combined medical and old-age care services in Jinjiang District, Chengdu. According to the Anderson model and the opinions of statistical experts, a questionnaire was designed, implemented in Questionnaire Star, and distributed to the residents of the Jinjiang District of Chengdu. Data were entered into Excel, and SPSS 27 was used to obtain descriptive statistics and to conduct single factor and logistic regression analyses. Statistically significant variables associated with older adults’ willingness to pay for combined services (*P* < .05) were age, occupation, education level, whether having commercial pension insurance, and whether there were health lectures in the community. There is high willingness to pay for combined medical and old-age care services in Jinjiang District.

## 1. Introduction

Combined medical and old-age care services represent the integration of resources, including diagnosis and treatment, nursing, rehabilitation treatment, physical examinations, health consultation, life care, mental and psychological therapy, cultural and recreational activities, and hospice care.^[1]^ China family structure has changed to a 4-2-1 hierarchy (four grandparents, 2 parents, and 1 child), which has exacerbated the burden of care for older persons of individual families and society.^[2]^ It has been reported that the number of people aged 80 and above in China is increasing at a rate of 4.7% annually and is expected to reach 25% to 30%.^[3]^ In 2020, the number of older persons with disabilities reached 52.71 million and is expected to exceed 77 million by 2030.^[4]^ The number of patients with dementia aged 60 and above reached 15.07 million.^[5]^ Chengdu has a total registered population of 12.1041 million, among which 2.4928 million are aged 60 and above, accounting for 20.60% of the total population.^[6]^ The demand for integrated medical and elder care services among community-based older people in Chengdu is relatively high.^[7]^ However, mismatch between supply and demand in the elder care industry is prominent.^[8]^ To date, the willingness of residents in Jinjiang District, Chengdu City, to pay for combined medical and elder care services (CMOCS) has not been investigated. Accordingly, we investigated this issue in the current study.

## 2. Methods

### 2.1. Research methods

Andersen et al^[9]^ proposed a health service utilization model – Andersen Behavioral Model – which is recognized in the industry as the best model to analyze factors that affect health service and medical mode selection behavior. The model can comprehensively analyze individual needs from a demand side perspective.^[10]^ This model has been adopted in many domestic studies of pension intention and has demonstrated efficacy in application.^[11–13]^ Based on a sample estimation method,^[14]^ the required sample size of this study was calculated as 341 cases. A questionnaire survey was conducted among randomly selected residents in Jinjiang District of Chengdu, using convenience sampling. The data collection tool was a 2-dimensional questionnaire survey. Residents were recruited through WeChat groups. After being informed of the study details, they voluntarily completed the questionnaire, which queried their willingness to pay for CMOCS. The inclusion criteria were permanent residents of Jinjiang District, with no mental illness, and who provided informed consent to complete the questionnaire. The exclusion criteria were nonresidents of Jinjiang District, those with poor communication ability, and older adults with dementia. Instructions were provided before completing the questionnaire. Quality control measures were implemented, such as error correction by 2 people and elimination of poor-quality completed questionnaires.

### 2.2. Statistical methods

The survey data were exported through Questionnaire Star, sorted using Excel, and analyzed using SPSS 27.0. The demographic data were described using frequencies and percentages, the χ^2^ test was used to compare differences between groups, Pearson contingency correlation analysis was used to assess the strength of the relationship between factors and willingness to pay, and the Freeman-Halton test was used to correct contingency tables that did not meet the Chi-square test requirements. According to the Andersen Behavioral Model, various factors that may affect willingness to pay for CMOCS were considered as independent variables. According to the theoretical analysis framework of the model, the independent variables were divided into 3 categories, as follows. Propensity factors included demographic characteristics, social structure, and factors related to the concept of old-age care, namely, gender, age, marital status, educational level, living situation, occupation, and propensity choice of old-age care mode. Enabling factors included the number of children, type of medical insurance, long-term care insurance, type of pension insurance, family’s choice of pension mode, whether they had purchased basic pension insurance, whether they had purchased basic medical insurance, whether they had purchased commercial pension insurance, and whether there were health lectures in the community. Demand factors included self-care ability, chronic disease presence, and chronic disease type. Multiple logistic regression was used to analyze the factors associated with residents’ willingness to pay. *P* <.05 was considered statistically significant.

## 3. Results

### 3.1. Survey respondents’ knowledge of CMOCS

A total of 341 questionnaires were distributed and 337 were collected, yielding an effective recovery rate of 98% (Table 1). Of the respondents, 42.44% expressed high awareness of this mode of care, whereas 57.56% of them were unaware of the mode of CMOCS.

**Table 1:** Basic characteristics of the respondents (n = 337 people).

Item	Characteristics of survey objects	Number of cases	Composition ratio/%
Sex	Male	89	26.41
	Female	248	73.59
Age (year)	20–30	53	15.73
	31–45	194	57.57
	46–60	79	23.44
	61 and above	11	3.26
Occupation	Teacher	20	5.93
	Medical worker	161	47.77
	General employees, self-employed	76	22.55
	Peasant	6	1.78
	Civil servants, government workers	24	7.12
	other	50	14.84
Educational level	Primary and below	4	1.19
	Junior high school	11	3.26
	High school/technical secondary school	31	9.20
	Junior college	69	20.47
	Bachelor degree or above	222	65.88
Medical insurance (other than commercial insurance)	Have health insurance	326	96.74
	Without health insurance	11	3.26
Have you purchased commercial insurance?	There are	196	58.16
	none	141	41.84
Are there any chronic diseases?	There are	34	10.09
	none	303	89.91
Marital status	mateless	60	17.80
	Have a spouse	277	82.20
Place of residence	city	322	95.55
	village	15	4.45
Number of children	childlessness	61	18.10
	one	186	55.19
	two	85	25.22
	Three or more	5	1.48
Availability of medical services in the community	There are	274	81.31
	None	63	18.69

### 3.2. Respondents’ willingness to choose CMOCS

Most (51.34%) of the survey respondents tended to choose home care for older adults, followed by those who chose home + community care for older adults (22.26%), community care for older adults (9.50%), and finally, institutional care for older adults (16.91%).

### 3.3. Factors associated with respondents’ willingness to pay for CMOCS

#### 3.3.1. Single-factor analysis of respondents’ willingness to pay

**Figure 1.**
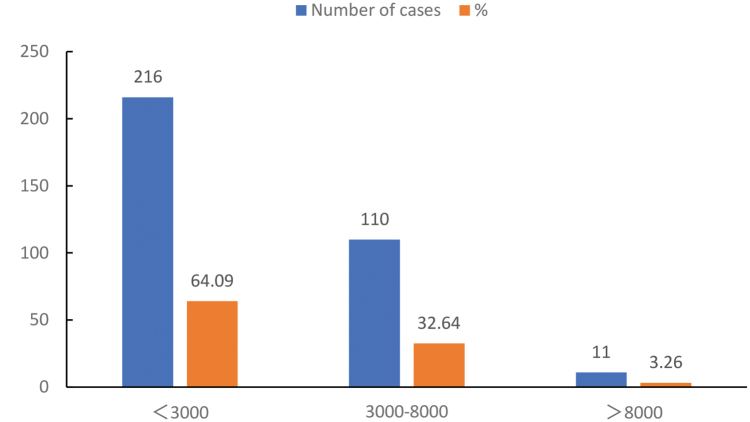
Distribution of respondents’ willingness to pay for the combination of medical care and old-age care.

Most of the respondents (64.09%) were willing to pay less than 3000 yuan/month, followed by 32.64% who were willing to pay between 3000 yuan and 8000 yuan/month, and 3.26% who were willing to pay more than 8000 yuan/month (Fig. 1). Comparing the propensity factors, enabling factors, and demand factors of residents with different payment intentions, the results showed that 3 propensity factors, whether having commercial pension insurance, and whether health lectures were conducted in the community influenced the residents’ intention to pay for CMOCS (*P* < .05; Table 2).

**Table 2:** Single-factor analysis of residents’ willingness to pay with different characteristics (n [%]).

Variable	Willingness to pay			*x* ^2^	*P*
	<3000	3000–8000	>8000		
Sex					
male	53 (24.5)	30 (27.3)	6 (54.5)	4.912	.086
female	163 (75.5)	80 (72.7)	5 (45.5)		
Age (years)					
20–30	41 (19.0)	9 (8.2)	3 (27.3)	11.554	.038
31–45	121 (56.0)	67 (60.9)	6 (54.5)		
46–60	46 (21.3)	32 (29.1)	1 (9.1)		
61 and above	8 (3.7)	2 (1.8)	1 (9.1)		
Occupation					
Teacher	10 (4.6)	10 (9.1)	0 (0)	24.741	.006
Medical worker	105 (48.6)	56 (50.9)	0 (0)		
General employees, self-employed	50 (23.1)	20 (18.2)	6 (54.5)		
Peasant	5 (2.3)	0 (0)	1 (9.1)		
Civil servants, government workers	15 (6.9)	9 (8.2)	0 (0)		
other	3 (14.4)	15 (14.6)	4 (36.4)		
Educational level					
Primary and below	3 (1.4)	0 (0)	1 (9.1)	18.057	.021
Junior high school	10 (4.6)	0 (0)	1 (9.1)		
High school/technical secondary school	2 (10.2)	9 (8.2)	0 (0)		
Junior college	46 (21.3)	23 (20.9)	0 (0)		
Bachelor degree or above	135 (62.5)	78 (70.9)	9 (81.8)		
Medical insurance					
Have health insurance	206 (95.4)	109 (99.1)	11 (100)	3.579	.167
None	10 (4.6)	1 (0.9)	0 (0)		
Have you purchased commercial insurance?					
There are	106 (49.1)	79 (71.8)	11 (100)	23.674	<.001
None	110 (50.9)	31 (28.2)	0 (0)		
Are there any chronic diseases?					
There are	24 (11.1)	10 (9.1)	0 (0)	1.604	.448
None	192 (88.9)	100 (90.9)	11 (100)		
Marital status					
mateless	40 (18.5)	16 (14.5)	4 (36.4)	3.463	.177
Have a spouse	176 (81.5)	94 (85.5)	7 (63.6)		
Place of Residence					
city	202 (93.5)	109 (99.1)	11 (100)	5.851	.054
village	14 (6.5)	1 (0.9)	0 (0)		
Number of children					
Childlessness	38 (17.6)	19 (17.3)	4 (36.4)	6.303	.390
One	122 (56.5)	60 (54.5)	4 (36.4)		
Two	51 (23.6)	31 (28.2)	3 (27.3)		
Three or more	5 (2.3)	0 (0)	0 (0)		
Availability of medical services in the community					
There are	168 (77.8)	96 (87.3)	10 (90.9)	5.013	.082
none	48 (22.2)	14 (12.7)	1 (9.1)		
Are there health seminars in the community?					
There are	105 (48.6)	63 (57.3)	10 (90.9)	8.814	.012
none	111 (51.4)	47 (42.7)	1 (9.1)		
How much do you know about the combination of medical and nursing care?					
Fully understand	55 (25.5)	38 (34.5)	3 (27.3)	7.863	.248
General understanding	26 (12.0)	19 (17.3)	2 (18.2)		
Have some understanding of	121 (56.0)	50 (45.5)	6 (54.5)		
Not a clue	14 (6.5)	3 (2.7)	0 (0)		
Choice of pension mode					
Home care for the elderly	115 (53.2)	49 (44.5)	9 (81.8)	8.083	.232
Community elderly care	21 (9.7)	11 (10)	0 (0)		
Institutional pension	34 (15.7)	21 (19.1)	2 (18.2)		
Home care + community care	46 (21.3)	29 (26.4)	0 (0)		

#### 3.3.2. Multi-classification logistic regression analysis of pension payment intention

A multi-factor logistic regression model was tested with residents’ willingness to pay as the dependent variable and variables with statistically significant differences in the univariate analysis as independent variables. The logistic regression results (Table 3) showed that residents’ willingness to choose and pay for CMOCS was related to age (odds ratio (OR) for 31–45 years = 2.73), education level (OR for civil servants or government workers = 1.07), and whether to holding commercial pension insurance (OR = 0.21).

**Table 3:** Multi-classification logistic regression analysis of residents’ willingness to pay for the combination of medical care and old-age care in Jinjiang District of Chengdu.

Variable		<3000		3000–8000	
		OR(95% CI)	*P*	OR(95% CI)	*P*
Age (years)	20–30	1.68 (1. 19– 2. 38)	.018	1. 20 (1.07–2.38)	.049
	31–45	2. 73 (1.85– 3. 34)	.014	2.89 (1.65–2.56)	.017
	46–60	2. 07 (1.32– 3. 57)	.019	1.79 (1.34–4.48)	.044
	61 and above	1.00		1.00	
Occupation	Teacher	1.09 (0.67–1.81)	.113	1.82 (0.34–4.63)	.485
	Medical worker	1.04 (0.72–1.50)	.051	1.59 (0.99–2.58)	. 460
	General employees, self-employed	0.57 (0.05–6.33)	.839	0.56 (0.15–7.02)	. 233
	Peasant	1.24 (0.80–1.93)	.769	1.92 (0.34–2.78)	. 350
	Civil servants, government workers	1.07 (0.65–1.77)	.041	1.33 (0.39–3.88)	. 135
	other	1.00		1.00	
Educational level	Primary and below	3.60 (1.74–7.43)	.021	2.14 (1.52–6.83)	.034
	Junior high school	2.77 (1.17–5.60)	.026	2.17 (1.71–3.16)	.005
	High school/technical secondary school	3.39 (1.52–6.56)	.041	2.35 (1.72–3.20)	.044
	Junior college	3.10 (1.36–7.05)	.034	3. 77 (2.14–6.64)	.135
	Bachelor degree or above	1.00		1.00	
Have you purchased commercial insurance?	There are	0.21 (0.12–0.38)	.030	0.57 (0.05–0.93)	<.001
	None	1.00		1.00	
Are there health seminars in the community?	There are	0.53 (0.05–6.02)	.395	0.78 (0.07–4.01)	.099
	None	1.00		1.00	

## 4. Discussion

### 4.1. Analysis of survey respondents’ knowledge of CMOCS

Compared with Kan et al^[15]^ analysis of the cognition status and factors that influence the willingness of middle-aged and older adults in Chengdu to CMOCS in 2017, their study showed that 84.9% of middle-aged and older adults in Chengdu did not know about CMOCS in 2017. In the current study, 57.56% were unaware of the mode of CMOCS, and 81.01% of residents in Jinjiang District were aged 31 to 60 years old. Therefore, the policy of CMOCS should be further publicized for residents of different ages. This would facilitate their choice of this mode of care.

### 4.2. Survey respondents’ choice, and analysis of the mode of CMOCS

Sweden elder care service concept advocates that older people live at home for a longer period of time,^[16]^ while Japan approach is a combination of family, community, and institution-based care.^[17]^ In this study, the proportion who desired home care was 51%, 22% preferred home and community care, 10% preferred community care, and 17% chose institutional care. In 2017, a study in Chengdu showed that 91.1% of residents chose self-care and family-based elder care, while only 8.9% chose community and institutional elder care. The factors that influence community residents in Chengdu to choose the integrated medical and elder care model included educational level, marital status, whether they had chronic diseases, elder care methods, and understanding of such services.^[15]^

### 4.3. Analysis of respondents’ willingness to pay for CMOCS

This study shows that the respondents’ willingness to pay for CMOCS is affected by factors such as age, educational level, and whether they hold commercial insurance. Some studies also showed that a higher level of education can significantly enhance the willingness of older people to pay for integrated medical and elder care services.^[18]^ A survey of the willingness to pay for CMOCS in Lanzhou City showed that this is related to differences in the needs, ages, and lifestyles of the older persons.^[19]^ The willingness of rural older people in Shandong Province to provide for their old age is significantly influenced by personal choice factors.^[20]^ According to a 2017 survey in Chengdu, the willingness to pay for CMOCS was low, with the most frequent acceptable expense being less than 2000 yuan per month. However, in the current study, more respondents were willing to pay up to 3000 yuan per month. The willingness to pay was affected by factors such as age, educational level, and whether commercial insurance was purchased. The differences between the results of this and the aforementioned studies may be related to the geographical location and age of the participants, differences in the surveyed population, different survey times, and the intervening promotion of the CMOCS.

Of the residents in Jinjiang District, 64.09% were willing to pay less than 3000 yuan per month. Community and family care were the most favored forms of elder care for the residents in Jinjiang District, Chengdu City. There exists potential demand for CMOCS among residents of Jinjiang District, many of whom are willing and able to pay for it. However, for this potential demand to become actual effective demand, a medical security system and an independent medical insurance fund need to be established, and a long-term care insurance fund system to coordinate mutual relief and maintain the minimum dignity of older adults is necessary.^[21]^

## 5. Conclusions

There is high willingness to pay for CMOCS in Jinjiang District. This willingness is affected by age, education level, and whether the individual holds commercial insurance.

Supplemental digital content “Supplementary-Questionnaire survey on residents” is available for this article (https://links.lww.com/MD/Q324).

## Acknowledgments

The authors thank Dr Yangfeng Huang for assistance with data extraction.

## Author contributions

**Conceptualization:** Chaorong Guo.

**Data curation:** Chaorong Guo, Ye Zhao.

**Formal analysis:** Chaorong Guo, Yongguang Lu.

**Investigation:** Yongguang Lu, Hui Zhu.

**Methodology:** Yongguang Lu, Hui Zhu.

**Project administration:** Hui Zhu.

**Resources:** Hui Zhu, Ye Zhao.

**Software:** Hui Zhu.

**Supervision:** Ye Zhao.

**Validation:** Yongguang Lu, Ye Zhao.

**Visualization:** Yongguang Lu, Hui Zhu.

**Writing – original draft:** Chaorong Guo, Yongguang Lu, Hui Zhu, Ye Zhao.

**Writing – review & editing:** Yongguang Lu, Hui Zhu, Ye Zhao.

## Supplementary Material



## References

[1] Qin S Cheng Y Zhang H Ding Y. Home/community based medical and elderly care services utilization in China: a cross-sectional study from the middle-aged and elderly population. Healthcare (Basel). 2023;11:2431. 37685465 10.3390/healthcare11172431 PMC10486956

[2] Jiang Q Sánchez-Barricarte JJ. The 4-2-1 family structure in China: a survival analysis based on life tables. Eur J Ageing. 2011;8:119. 28798645 10.1007/s10433-011-0189-1 PMC5547295

[3] Xie Y Zhao Q Xiao M. The current status of integrated medical and nursing service in Chongqing. Chin Nurs Manag. 2022;22:548–52.

[4] Luo Y Su B Zheng X. Trends and challenges for population and health during population aging - China, 2015-2050. China CDC Wkly. 2021;3:593–8. 34594944 10.46234/ccdcw2021.158 PMC8393078

[5] Jia LF Du YF Chu L. Prevalence, risk factors, and management of dementia and mild cognitive impairment in adults aged 60 years or older in China: a cross-sectional study. Lancet Public Health. 2020;5:e661–71. 33271079 10.1016/S2468-2667(20)30185-7

[6] Wang Y Tian F Fan N Pan J. Elderly resident’s awareness, attitude and willingness-to-pay for elderly care medical institutions: an empirical analysis based on Chengdu. Chin J Health Policy. 2017;10:18–22.

[7] Qin L Wen Z Kun S. Status Quo and its influencing factors of the demand for integration of medical treatment and care service of home–based elderly in the community of Chengdu City. Med Soc. 2020;33:65–68 + 74.

[8] Du N Wu P Yuan M Li Z. Performance evaluation of combining with medical and old-age care in pension institutions of China: a two-stage data envelopment analysis. Risk Manag Healthc Policy. 2021;14:4211–22. Erratum in: Risk Manag Healthc Policy. 2025;18:1513–4. 34675715 10.2147/RMHP.S332880 PMC8518464

[9] Andersen R Newman JF. Societal and individual determinants of medical care utilization in the United States. Milbank Mem Fund Q Health Soc. 1973;51:95–124. 4198894

[10] Alkhawaldeh A ALBashtawy M Rayan A. Application and use of Andersen’s behavioral model as theoretical framework: a systematic literature review from 2012-2021. Iran J Public Health. 2023;52:1346–54. 37593505 10.18502/ijph.v52i7.13236 PMC10430393

[11] Zhang Y Wang L. Family care and predictors of the disabled elderly in China: a cross-sectional study based on the Anderson model. PLoS One. 2024;19:e0312002. 39495768 10.1371/journal.pone.0312002 PMC11534204

[12] Zeng L Xu X Zhang C Chen L. Factors influencing long-term care service needs among the elderly based on the latest Anderson model: a case study from the middle and upper reaches of the Yangtze River. Healthcare (Basel). 2019;7:157. 31816957 10.3390/healthcare7040157 PMC6955999

[13] Evashwick C Rowe G Diehr P Branch L. Factors explaining the use of health care services by the elderly. Health Serv Res. 1984;19:357–82. 6746297 PMC1068819

[14] Sun J Chen W Wang J. Therapeutic effect of tamsulosin combined with escitalopram in the treatment of chronic pelvic pain syndrome. Hebei Med J. 2024;46:2462–5.

[15] Wu K Cao PY Qian JH Luo HQ Liu DP. Perceptions and attitudes of Chengdu residents toward “medical and aged care” integrated models. J Sichuan Univ (Med Sci Ed). 2017;48:455–9. 28616925

[16] Österholm J Olaison A Larsson AT. Age-appropriate elder care recipients? Care manager’s categorisation practices in intraprofessional case conferences. J Aging Stud. 2024;69:101234. 38834254 10.1016/j.jaging.2024.101234

[17] Chang SH. New directions in elderly care workforce nurturing: cross-professional collaboration from a social co-care perspective, drawing insights from Japan’s elderly care experience. Hu Li Za Zhi. 2025;72:22–8. Chinese. 39838791 10.6224/JN.202502_72(1).04

[18] Wei Y Sun Y Li Y. Investigation and research on elderly people’s willingness to combine medical and health care and related factors in coastal cities in eastern China. PeerJ. 2022;10:e14004. 36097524 10.7717/peerj.14004 PMC9463997

[19] Wang J Wang Y Cai H. Analysis of the status quo of the elderly’s demands of medical and elderly care combination in the underdeveloped regions of Western China and its influencing factors: a case study of Lanzhou. BMC Geriatr. 2020;20:338. 32907557 10.1186/s12877-020-01616-6 PMC7488146

[20] Xu X Li P Ampon-Wireko S. The willingness and influencing factors to choose institutional elder care among rural elderly: an empirical analysis based on the survey data of Shandong Province. BMC Geriatr. 2024;24:17. 38177989 10.1186/s12877-023-04615-5 PMC10768132

[21] Zhou Y-R Zhang X. The experience and enlightenment of the community-based long-term care in Japan. Healthcare (Basel). 2022;10:1599. 36141211 10.3390/healthcare10091599 PMC9498550

