# Supplementary material for: Willingness to pay for combined medical and old-age care services: A survey in Chengdu City, China
Source: Medicine (Baltimore). 2025 Oct 17;104(42):e45206. doi: 10.1097/MD.0000000000045206 (PMC12537190; doi:10.1097/MD.0000000000045206)
Supplement: Supplementary file 1 [file medi-104-e45206-s001.docx]

**Questionnaire survey on residents' willingness to pay for the combination of medical and nursing care in Jinjiang District**

Dear residents of Jinjiang District of Chengdu：

Hello! Now a survey is conducted on the willingness to pay for the combination of medical and old-age care mode, which is limited to public health scientific investigation and research, anonymous filling, and will not disclose personal information! Thank you for your cooperation and understanding！

| Item | Options | | | | | |
| --- | --- | --- | --- | --- | --- | --- |
| Sex | Male | ② Female | | | | |
| Age | ①20-30 | ②31-45 | ③46-60 | ④≥61 | | |
| Occupation | ① Teacher | ② Medical worker | ③ Company staff、 Manual worker | ④ Peasant | ⑤ Civil servants, government workers | ⑥ Other |
| Educational level | ① Primary and below | ② Junior high school | ③ Secondary school/high school | ④ Junior college | ⑤ Bachelor degree or above | |
| Health insurance or not | ① Has health insurance | ② No health insurance | | | | |
| Whether to purchase commercial insurance | ① purchase commercial insurance | ② No commercial insurance | | | | |
| Are there health talks in the community? | ①yes | ②Are not | | | | |
| Any chronic diseases? | ① yes | ②no | | | | |
| Marital status | ① Unmarried | ② Married | ③ Divorce/widowhood | | | |
| Place of residence | ①City | ② Village | | | | |
| Number of children | ① progeny | ②1 | ③2 | ④≥3 | | |
| Is there medical care in the community? | ①yes | ②is not | | | | |
| Cognition degree of medical care combined with elderly care services | ① Very clear | ② clearer | ③ Know a bit about | ④ Know nothing about | | |
| Choice of pension mode | ①Home care for the elderly | ②Community elderly care | ③Institutional pension | ④Home care + community care | | |
|  | ① Very important | ② Of great importance | ③ importance | ④ Less important | ⑤ insignificance | |
| Whether the pension institution can be reimbursed by medical insurance |  |  |  |  |  |  |
| The older-adult care institution has medical facilities, doctors, and nurses |  |  |  |  |  |  |
| Whether the older-adult care institution has a cooperative medical unit |  |  |  |  |  |  |
| Is there a hospital near the nursing home |  |  |  |  |  |  |
| Whether the elderly care institution has a green environment |  |  |  |  |  |  |
| Accommodation and diet in older-adult care institutions |  |  |  |  |  |  |
| Entertainment culture in older-adult care institutions |  |  |  |  |  |  |
| Medical care, health education, rehabilitation treatment |  |  |  |  |  |  |
| Quality of medical care in nursing institutions |  |  |  |  |  |  |
| Quality of life care service in nursing institutions |  |  |  |  |  |  |
| Service qualification and quality of nursing workers in older-adult care institutions |  |  |  |  |  |  |
| There are volunteers and social workers in the nursing home |  |  |  |  |  |  |
| Charge standard for older-adult care institutions | ①3000 yuan/month | ②3000-8000 yuan/month | ③＞8000 yuan/month | | | |
